# Current Status of Phlebotomy Training Among Novice Nurses in Japan: A Survey Analysis

**DOI:** 10.7759/cureus.28100

**Published:** 2022-08-17

**Authors:** Yukari Shimizu, Etsuko Matsumura, Sachiko Tachibana

**Affiliations:** 1 Department of Nursing, Komatsu University, Komatsu, JPN; 2 Department of Nursing, Fukui Health Sciences University, Fukui, JPN

**Keywords:** text mining, patient role, nurse role, phlebotomy, novice nurses

## Abstract

Introduction

Phlebotomy is an invasive technique that requires technical competence, which often makes novice nurses nervous. We created a phlebotomy training program with roleplaying scenarios in which the learners played the role of a nurse and a patient.

Methods

The study included 84 novice nurses. They played the role of a patient and a nurse and were administered a survey at the end of the training. Data were analyzed using text mining and natural language processing.

Results

By playing the role of a patient, novice nurses were able to increase awareness of patients’ feelings and understand the principles of nursing techniques.

Conclusions

Phlebotomy training involving roleplay enabled learners to understand the importance of communication and how to make their patients feel comfortable.

## Introduction

In Japan, phlebotomy is almost exclusively performed by the nursing staff although a subset is performed by medical laboratory technologists. In contrast, these clinical tasks are delegated to specific medical professionals in other countries such as the US and UK [1]. For example, training is sufficient for an individual to become a phlebotomist, i.e. a medical assistant that performs phlebotomy, and they do not require any other medical certification or degree. Studies have demonstrated that the advantage of having phlebotomists is that medical professionals are able to focus on tasks that require advanced medical skills.

Nursing practice has become diverse due to advancements in medicine, changes in the types of diseases, aging of the patient population, and reduced lengths of stay. Nurses have a significant role in addressing and adapting to these changes and expectations of the general public regarding the level of medical care expected. Novice nurses are also required to meet such expectations; thus, it is critical to enhance the skills of novice nurses to ensure the delivery of safe and high-quality care.

However, there is significant variation among different nursing curriculums. The Ministry of Health, Labour and Welfare reported that there is a discrepancy between the technical skills of graduating nurses and the expected level of competency in the clinical setting [2]. Notably, since nursing students do not have a nursing license, they are limited in terms of the types of technical skills they can learn while in the clinical setting.

At the Fukui University Hospital, we conducted hands-on training for novice nurses who had just started their jobs in April in order to enhance their practical skills. Additionally, as part of the effort led by the prefecture, we also invited novice nurses working in small-to-medium, non-academic hospitals or clinics to improve their overall quality and skills.

The training was structured with individual courses that had specific purposes and learning objectives and used clinical equipment and tools to simulate the clinical setting. As learners find their positions in the hospitals and clinics, we administered a survey to ask them about the skills that they feel uncomfortable performing so that we could plan our training to focus on those skills.

Our training focused on phlebotomy since it is a high-priority skill required in the clinical setting that novice nurses often feel uncomfortable performing. Since phlebotomy is highly invasive, it is important to ensure that the procedure is performed safely and, more importantly, to ensure that patients feel comfortable while undergoing the procedure [3].

As students, we have been taught that "venipuncture should generally be performed in the superficial vein (cephalic, basilic, and medial cubital veins in the anterior elbow) but can also be performed in the dorsal hand veins if the former is challenging [4]." Thus, we confirmed that those who played the role of a nurse asked the patients if they have any preferences in terms of the site of venipuncture.

Subjects who played the role of nurses communicated with those who played the role of patients in order to reduce their anxiety. For example, they inform the patients that they are starting the blood collection. Prior to phlebotomy, they told the patients to make a fist and explained where venipuncture will be performed and how much blood (in mL) will be drawn. They maintained eye contact with the patient throughout the process. During phlebotomy, they made sure that the patients were not experiencing any pain or numbness in their hands. At the end of phlebotomy, they told the patients that the needle will be withdrawn and that they need to keep pressing the site of venipuncture for three to five minutes to stop the bleeding and to keep the bandage for the day.

Hirata et al. demonstrated that hands-on training of basic nursing skills that involve learners to play the role of both nurse and patient was effective in recognizing their role as nurses and improved their awareness of patients’ feelings [5]. Furthermore, Kuribayashi demonstrated that the experience of being an in-patient as part of the role-play enhanced the ability of learners to imagine what patients may feel while receiving care and to better understand the principles of nursing techniques (i.e., why a technique is performed in a particular way) [6].

There are several studies on phlebotomy that examined the perception of nursing students on role-play as a patient in phlebotomy training [7-10]. However, there is a lack of studies that examine the thought processes of novice nurses while performing the roles of nurse or patient during such training. In the present study, we analyzed the thought processes of novice nurses playing the role of a nurse and patient as part of phlebotomy training. Specifically, we analyzed the survey submitted after the training session to understand the learning experience the roleplay had provided to the learners. The information provided by the study should help improve the effectiveness of phlebotomy training for nurses.

The present study aimed to understand the thought processes of novice nurses while playing the role of a patient and nurse during phlebotomy training.

## Materials and methods

Study participants and ethical considerations

The study included 84 nursing graduates who started their job at the Fukui hospital in 2013. The study duration was from April 11, 2013 to April 18, 2013. Their mean age was 21.9 ± 2.4 years, and there were 79 females and five males. Their education level was high school with nursing specialization (n = 1, 1.3%), nursing school (n = 22, 26%), junior college (n = 16, 19%), and university (n = 45, 53.7%).

Ethical approval for this study was obtained from the Ethics Committee of the Department of Nursing, University of Fukui Hospital, approval number 1901. Participants were provided verbal and written explanation of the purpose of the study and its privacy policy. They were informed that their participation was voluntary and will not affect their job responsibilities, that the data will be aggregated for statistical analysis, and the results will not include personal information. Signed consent was obtained prior to participation in the study.

Characteristics of study participants 

All 84 participants returned their completed survey. Participants included 79 women (95%) and five men (5%), with a mean age of 21.9 ± 2.4 years. Education degree of participants included university (n = 45, 53.7%), nursing school (n = 22, 26%), junior college (n = 16, 19%), and high school with nursing specialization (n = 1, 1.3%).

Survey

An anonymized survey was distributed prior to the start of phlebotomy training. After participating in a training session that involved them playing the role of patient and nurse during phlebotomy, participants used the survey to describe what they felt while playing those roles. The surveys that were submitted by the participants after playing the role of patient and nurse included a total of 1,204 terms.

Analysis

A text-mining software (Text Mining Studio Ver 5.0, NTT) was used for the analysis. All participants used Microsoft Word 2011 to submit the survey with their comments, and the comments were run through into the software to perform term frequency analysis, dependency parsing, and network analysis (co-occurrence analysis) to determine the differences in the thoughts of nurses playing the role of a patient versus a nurse [11].

## Results

Term frequency analysis

In the present study, we performed the frequency analysis of the terms used by a total of 82 subjects who played the roles of nurses and patients. We then performed additional analysis on the use of the terms “think” and “needle” as these terms were identified as having co-occurrence.

**Figure 1:**
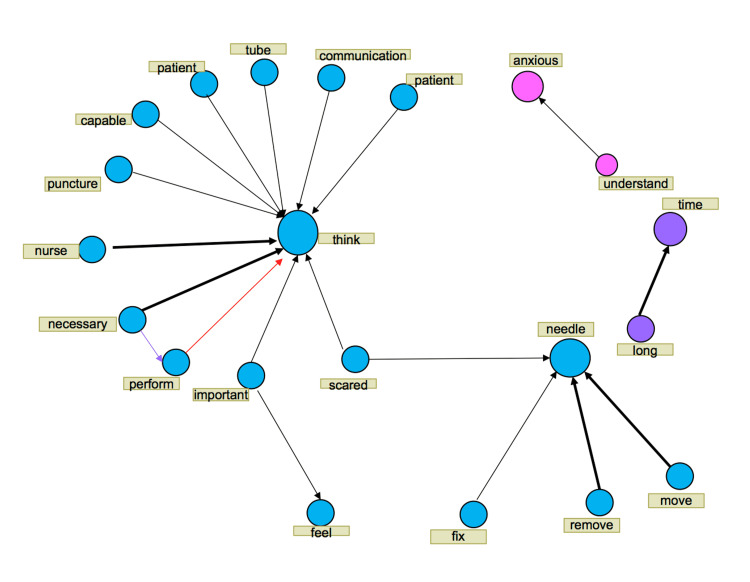
Text network (role of a patient during phlebotomy)

Terms that were used to describe the experience of playing the role of a patient and their frequency of use, indicated as “term” (frequency), were “think” (67), “needle” (49), “anxious” (40), “feel” (32), “communication” (32), “puncture” (27), “draw” (25), “patient” (24), and “nervous” (23) (Figure 1).

**Figure 2:**
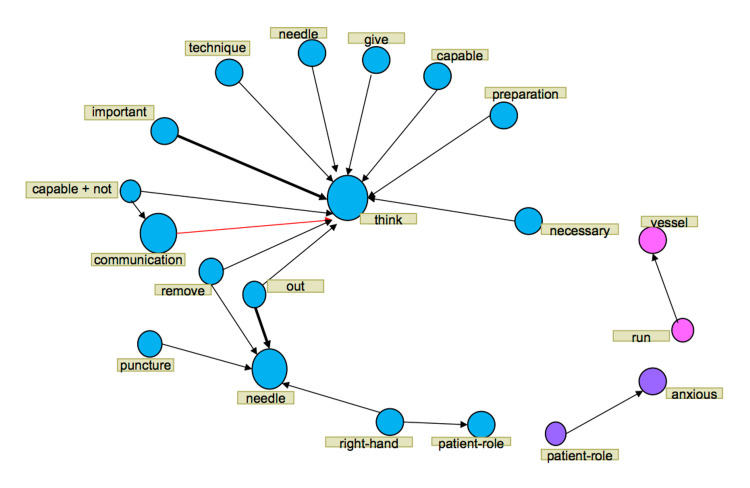
Text network (role of a nurse during phlebotomy)

Similarly, terms that were used to describe the experience of playing the role of a nurse and their frequency of use were “think” (73), “needle” (59), “tube” (42), “fix” (42), “vessel” (40), “communication” (40), “patient” (36), “hand” (29), “draw” (28), “anxious” (27), “puncture” (25), and “nervous” (23) (Figure 2).

Text network

Additional analysis was performed on terms with co-occurrence, specifically “think” and “needle”.

For the role of a patient, “think” was interconnected to “patient”, “communication”, “tube”, and “capable”, and “needle” was interconnected to “move”, “out”, “fix”, and “scared”.

For the role of a nurse, “think” was interconnected to “necessary”, “preparation”, “capable”, and “give”, and “needle” was interconnected to “right-hand”, “puncture”, and “out”.

## Discussion

In the present study, we analyzed the response from the survey that was submitted by novice nurses following the phlebotomy training in which they played the role of both patient and nurse to determine the difference in their experiences in each role. Results from the term frequency and co-occurrence analyses demonstrated that participants described the role of a patient with terms related to communication and technique, particularly those that are related to emotions and experiences such as "nervous", "pain", "hurt", "good", and "scared". Similarly, Kuribayashi demonstrated that experiencing the role of a patient enhanced the ability of nurses to imagine what patients may feel while receiving care and to better understand the principles of nursing techniques (i.e., why a technique is performed a particular way) [6].

Nursing builds on human interaction, and nursing students learn basic communication skills and techniques throughout their training so that they are able to make patients feel comfortable while providing care [12,4]. Thus, novice nurses likely focus on the importance of communication as seen in our study.

Our findings also demonstrated that participants described the role of a nurse with terms such as preparation and those related to technical skills, including "needle", "tube", "fix", "vessel", "hand", and "puncture". This indicates that nurses used these terms to reduce the anxiety of both themselves and patients who are expecting accurate phlebotomy techniques from the nurses. Kawatsu and Nin performed a study on the general public, physicians, nurses, and nurse educators to identify the requirements of nurses to meet the expectation of their patients [13]. The authors performed a factor analysis of data collected from all participants and identified five factors; specifically, the average score for the "technicality" factor was ranked the highest among the general public included in the study (n = 54). In contrast, we demonstrated that participants in the role of a nurse were more likely to try not to make the procedure painful to patients rather than focusing on communicating with them during the procedure. This suggests that as students, novice nurses were taught to focus primarily on minimizing pain and discomfort for patients. In fact, the frequency of terms that were identified in the study likely reflected the training novice nurses had received in school, which primarily focused on the safety and comfort of, and respect for, patients. Our findings also indicated that the participants were reflecting on their experience while playing the role of both nurse and patient to identify specific methods of communication and assistance that would improve the safety and comfort of patients while performing nursing procedures.

## Conclusions

Term frequency analysis revealed that terms used frequently while in the role of a patient included “think”, “needle”, and “anxious” whereas those used while in the role of a nurse included “think”, “needle”, “tube”, and “fix”. In the co-occurrence analysis, we focused on the terms “think” and “needle”.

For the role of a patient, “think” was interconnected to “patient”, “communication”, “tube”, and “capable”, and “needle” was interconnected to “move”, “out”, “fix”, and “scared”. For the role of a nurse, “think” was interconnected to “necessary”, “preparation”, “capable”, and “give”, and “needle” was interconnected to “right-hand”, “puncture”, and “out”.

These findings indicate that while the nurse was primarily focused on the technique of phlebotomy, the patient was interested in both the technique and how the nurses communicated with them.

The results and conclusions of the study were thoroughly reviewed by the supervisors. We also noted that the way the subjects were trained on phlebotomy in terms of how the training was structured in the curriculum, what the training consisted of, and how many hours of training they received may differ due to the differences in the type of educational environment (i.e. university, junior college, technical college, major subject). Since we did not examine such factors of educational background in detail, we plan to incorporate them as part of variables of social background in future studies.
